# Lacerated Wound of the Scalp—Union by the First Intention

**Published:** 1838-08-29

**Authors:** J. M. Wallace

**Affiliations:** Late Resident Surgeon


*Lacerated wound of the scalp—union by the first intention.*

[Reported by J. M. WALLACE, M. D., late Resident Surgeon.]

ELIZABETH M——, aged fifty years, admitted March 17th, 1837. The corner of a wooden water trough fell upon her head, from a height of six feet; it struck her directly in the line of the sagittal suture, and glanced towards the right side; there was a wound of five inches from the top of the forehead directly backwards, and another from this point downwards to the ear. A flap, consisting of the skin and the occipito-frontalis muscle was torn off and hung over; the temporal artery was ruptured, and considerable hæmorrhage had taken place, before her admission, from that and several small vessels, which still bled freely. The hair was driven under the scalp; she had no symptoms of concussion, and complained merely of pain in the wound. The head was shaved and cold water allowed to run over the wounded vessels, the extremities of which were twisted. The bleeding was soon checked; the edges of the wound were drawn together by adhesive plaster, simple cerate, and a light bandage applied; cold drinks and cold gruel given.

*March 18th.*—No bad symptoms; complains of some heat in the head; cold lotions applied, and a purgative given.

*March 21st.*—Scalp examined to-day; the wound was found to have united by the first intention, except at the lower corner touched with nitrate of silver. She was allowed warm food to-day for the first time since the accident, but confined to a vegetable diet.

*March 27th.*—Discharged cured.

